# The role of vimentin in the tumor marker Nup88-dependent multinucleated phenotype

**DOI:** 10.1186/s12885-018-4454-y

**Published:** 2018-05-03

**Authors:** Masaki Makise, Hideaki Nakamura, Akihiko Kuniyasu

**Affiliations:** Faculty of Pharmaceutical Sciences, Sojo University, 4-22-1 Ikeda, Nishi-ku, Kumamoto, 860-0082 Japan

**Keywords:** Nup88, Nucleoporin, Vimentin, Cancer, Multinucleated phenotype, Kinases, Interaction

## Abstract

**Background:**

Nucleoporin Nup88, a component of nuclear pore complexes, is known to be overexpressed in several types of tumor tissue. The overexpression of Nup88 has been reported to promote the early step of tumorigenesis by inducing multinuclei in both HeLa cells and a mouse model. However, the molecular basis of how Nup88 leads to a multinucleated phenotype remains unclear because of a lack of information concerning its binding partners. In this study, we characterize a novel interaction between Nup88 and vimentin. We also examine the involvement of vimentin in the Nup88-dependent multinucleated phenotype.

**Methods:**

Cells overexpressing tagged versions of Nup88, vimentin and their truncations were used in this study. Coprecipitation and GST-pulldown assays were carried out to analyze protein-protein interactions. Vimentin knockdown by siRNA was performed to examine the functional role of the Nup88-vimentin interaction in cells. The phosphorylation status of vimentin was analyzed by immunoblotting using an antibody specific for its phosphorylation site.

**Results:**

Vimentin was identified as a Nup88 interacting partner, although it did not bind to other nucleoporins, such as Nup50, Nup214, and Nup358, in HeLa cell lysates. The N-terminal 541 amino acid residues of Nup88 was found to be responsible for its interaction with vimentin. Recombinant GST-tagged Nup88 bound to recombinant vimentin in a GST-pulldown assay. Although overexpression of Nup88 in HeLa cells was observed mainly at the nuclear rim and in the cytoplasm, colocalization with vimentin was only partially detected at or around the nuclear rim. Disruption of the Nup88-vimentin interaction by vimentin specific siRNA transfection suppressed the Nup88-dependent multinucleated phenotype. An excess amount of Nup88 in cell lysates inhibited the dephosphorylation of a serine residue (Ser83) within the vimentin N-terminal region even in the absence and presence of an exogenous phosphatase. The N-terminal 96 amino acid residues of vimentin interacted with both full-length and the N-terminal 541 residues of Nup88.

**Conclusions:**

Nup88 can affect the phosphorylation status of vimentin, which may contribute to the Nup88-dependent multinucleated phenotype through changing the organization of vimentin.

## Background

Nuclear pore complexes (NPCs) are channels that mediate bidirectional trafficking between the cytoplasm and the nucleus in eukaryotic cells. In vertebrates, multiple copies of approximately 30 polypeptides, termed nucleoporins (Nups), assemble to form NPCs [1, 2]. A single NPC exhibits an eight-fold symmetric cylinder-like structure that can be divided into three distinct regions [2, 3]. One such region is the central channel, which spans the inner and outer nuclear membrane. The other two regions are the nuclear basket and the cytoplasmic filaments, both of which are joined by the central channel. Approximately one-third of nucleoporins have a variety of phenylalanine-glycine (FG) repeats [3, 4]. FG repeat-containing Nups form unstructured barriers in the central channel, which are crucial for the selective trafficking of macromolecules [4, 5].

Nup88 is a non-FG Nup located on the cytoplasmic face of the NPC that participates in nuclear export by interacting with other FG Nups, such as Nup358 and Nup214 [3, 6–8]. Intriguingly, abnormally high levels of Nup88 has been detected in the cytoplasm of various tumor cells [3, 9, 10]. Furthermore, it has been reported that elevated Nup88 expression in tumor cells is correlated with tumor grade such as in colorectal cancer, breast cancer and hepatocellular carcinoma [11–15]. Consequently, it has been proposed that Nup88 might be used as a cancer prognostic and diagnostic marker [3].

Recent reports indicate that Nup88 overexpression is responsible, at least in part, for the early steps of tumorigenesis. For instance, overexpression of Nup88 in HeLa cells induces multipolar spindles and the appearance of multinucleated cells [16]. In addition, elevated Nup88 expression in a mouse model induces multinuclei by promoting the premitotic destruction of Polo-like kinase 1 (Plk1), a kinase required for bipolar spindle formation and chromosome segregation [17, 18]. However, the molecular basis of how Nup88 leads to a multinucleated phenotype is poorly understood because of a lack of information concerning its interacting partners [19].

Proteomic analysis reveals a variety of proteins that associate with NPCs [1]. One such protein is vimentin, which is classified as a type III intermediate filament (IF) protein abundant in motile mesenchymal cells, various types of tumors, and cancer cell lines [20]. Vimentin forms insoluble filamentous networks that distribute widely in the cytoplasm from the perinuclear region to the cell periphery. Phosphorylation of vimentin turns its insoluble filaments into a soluble or insoluble non-filamentous form [21–23]. This organizational change of vimentin endows the cells with mechanical resistance, allowing them to undergo morphological change and motility [20, 24–27].

Proteomic approaches indicate that the N-terminal region of vimentin contains many amino acid (aa) residues that are phosphorylated by a variety of kinases, such as mitotic kinases [20, 25, 28, 29]. For instance, phosphorylation by cyclin-dependent kinase 1 (Cdk1) promotes vimentin IF disassembly into non-filamentous oligomers. In addition, Cdk1 promotes Plk1 specific vimentin phosphorylation during early mitosis [30–32]. Both Rho-associated kinase and Aurora B kinase promote the segregation of vimentin IF cooperatively at the cleaved furrow from late mitosis to cytokinesis [30, 33, 34]. Although vimentin overexpression in cancer cells has been reported to be closely correlated with accelerated tumor growth, migration, and invasion, specific regulation of the phosphorylation status of vimentin during the development of cancer remains poorly understood [20].

In the present study, to gain insights into the molecular basis of how overexpressed Nup88 induces multinuclei in HeLa cells, we screened Nup88 interacting proteins using a proteomic approach. Here, we demonstrate that a novel interaction of Nup88 with vimentin can affect the phosphorylation status of vimentin. We also propose that the interaction between Nup88 and vimentin is involved in the Nup88-dependent multinucleated phenotype.

## Methods

### Plasmid constructs

Plasmids expressing GFP-tagged proteins under the control of a CMV promoter were generated by cloning their PCR products into pEGFP-N2 (Clontech, Mountain View, CA). The expression plasmids for FLAG-tagged proteins were constructed by replacing the *EGFP* genes in pEGFP-N2 with synthetic oligonucleotides encoding 3 x *FLAG*. A plasmid that expresses GST-fused Nup88 was constructed by inserting the *Nup88* gene downstream of the *GST* gene in pGEX4T-3 (GE Healthcare Life Sciences, Piscataway, NJ). A plasmid that expresses Halo tagged Nup214 or Nup358 was purchased from Kazusa Genome Technologies Inc. [35].

### Cell culture and stable cell lines

Cells were cultured in Dulbecco’s modified Eagle’s medium (DMEM) supplemented with 10% fetal bovine serum and penicillin/streptomycin at 37 °C in a humidified incubator supplied with 5% CO_2_.

T-REx HeLa cells containing a single Flp recombination site in the genome were established from HeLa R19 cells [36]. The T-REx HeLa stable cell lines were established according to the manufacturer’s instructions (Life Technologies, Carlsbad, CA). Briefly, the T-REx HeLa cells were cotransfected with pcDNA5/FRT/TO encoding an EGFP-tagged version of the genes of interest and pOG44 encoding the Flp recombinase. Following transfection, the cells were transferred into medium containing 100 μg/ml hygromycin B and 3 μg/ml blasticidin S to select for drug-resistant clones. The clones were collected by trypsinization and stored frozen in liquid N_2_ before use. Protein expression in T-REx HeLa cells was induced using medium containing 1 μg/ml doxycycline (DOX) for 24-48 h.

### GFP-coprecipitation assay

Coimmunoprecipitation assays for GFP-fused proteins were performed as previously described [37]. Cell lysates (500-1500 μg) prepared with NP-40 lysis buffer (10 mM Tris/HCl pH 7.5, 150 mM NaCl, 5 mM EDTA, 0.5% Nonidet P-40) were incubated with 5-20 μl of GFP-trap A beads (ChromoTek, Munich, Germany) at 4 °C for 15-120 min. After incubation, the beads were collected by centrifugation at 2000 rpm at 4 °C for 1 min and washed three times with lysis buffer. The proteins on the beads were eluted by incubation with 2.5× SDS sample buffer (0.156 M Tris/HCl pH 6.8, 5% SDS, 25% glycerol, 0.0125% bromophenol blue, 12.5% 2-mercaptoethanol) at 100 °C for 1 min and then analyzed by SDS-PAGE. Protein bands were visualized by staining with Coomassie brilliant blue (CBB). Proteins extracted from the gel were identified by mass analysis using matrix-assisted laser desorption time-of-flight mass spectrometry.

### Purification of GST fusion proteins

*E. coli* cells expressing GST and GST fusion protein were harvested by centrifugation. The cell pellet was resuspended in NP-40 lysis buffer supplemented with 1% Triton X-100 and appropriate protease inhibitors. Cells were lysed by sonication. The supernatant was collected after centrifugation and incubated with glutathione-Sepharose 4B beads at 4 °C for 120 min with rotation. Proteins bound to the beads were washed three times with NP-40 lysis buffer and then eluted with elution buffer (5% glycerol, 50 mM Tris-HCl pH 8.0, 20 mM reduced glutathione, 0.5% NP-40, 200 mM NaCl, 2.5 mM MgCl_2_, 1 mM PMSF, 0.1 mM dithiothreitol). Eluates were dialyzed overnight against NP-40 lysis buffer supplemented with appropriate protease inhibitors at 4 °C.

### GST-pulldown assay

Purified GST fusion protein was incubated with recombinant human vimentin purified from *E. coli* (PeproTech, Rocky Hill NJ) and glutathione Sepharose 4B beads in NP-40 lysis buffer at 4 °C for 120 min with rotation. The beads were washed three times with NP-40 lysis buffer. Proteins bound to the beads were eluted with 2.5× SDS sample buffer.

### Antibodies

Mouse monoclonal anti-human Nup88 (#611896) was purchased from BD Biosciences (Franklin Lakes, NJ). Rabbit polyclonal anti-GFP (#598), anti-αtubulin (#PM054), and anti-DDDDK (#PM020) were purchased from Medical & Biological Laboratories (Tokyo, Japan). Rabbit polyclonal anti-GFP (#ab290), mouse monoclonal anti-vimentin (#sc-6260), rabbit monoclonal anti-pS83 vimentin (#12569), rabbit polyclonal anti-phospho-Histone H3 (#06-570), and mouse monoclonal anti-HaloTag® (#G9211) were purchased from Abcam (Cambridge, UK), Santa Cruz Biotechnologies (Dallas, TX), Cell Signaling Technologies (Danvers MA), Merck Millipore (Burlington, MA), and Promega (Madison, WI), respectively. For immunoblotting, all antibodies were applied at a dilution of 1:1000. Antibodies were diluted according to the manufacturer’s instructions for indirect immunofluorescence.

### Immunoblotting

Cell lysates were prepared with either radio immunoprecipitation assay (RIPA) buffer (50 mM Tris/HCl pH 8.0, 150 mM NaCl, 5 mM EDTA, 1% Nonidet P-40, 0.5% sodium deoxycholate, 0.1% SDS) or NP-40 lysis buffer supplemented with appropriate protease inhibitors. Lysates were subjected to SDS-PAGE and proteins separated on the gel were transferred onto an Immobilon-P transfer membrane (Merck Millipore). The membrane was blocked with blocking buffer (5% skimmed milk, 0.05% Tween 20 in PBS) for 30 min at room temperature and then incubated with primary antibodies (diluted with 1% *w*/*v* skimmed milk, 0.05% Tween 20 in PBS) at 4 °C for more than 10 h. After incubation, the membrane was washed with wash buffer (0.05% Tween 20 in PBS) three times at 5 min intervals and then incubated with secondary antibodies conjugated with horseradish peroxidase (HRP) at 4 °C for 1 h. Chemiluminescence was generated by the addition of Luminate Crescendo Western HRP substrate (Merck Millipore) to the membrane and was detected using a LAS2000 Imaging analyzer (Fuji Film, Tokyo, Japan).

### siRNA transfection

Control siRNA and vimentin specific siRNA (#4427038) were purchased from Ambion (Foster City, CA). The transfection of siRNA was performed using RNAiMAX transfection reagent (Life Technologies). Briefly, 1 day before transfection, cells were plated at a density of 4 × 10^5^ cells per well in a 12-well plate with 1 ml of culture medium and then cultured until 80% confluent. siRNA was then incubated with the transfection reagent in a reduced serum medium for 25 min to form a lipid-siRNA complex, and then transfected into cells. After a 24 h incubation, the medium was exchanged with fresh pre-warmed medium. The cells were further incubated until immediately prior to analyses.

### Indirect immunofluorescence assay

Cells grown on coverslips in 24-well plates were washed once with pre-warmed PBS and then fixed with 4% paraformaldehyde for 15 min, followed by permeabilization with PBS containing 0.5% Triton X-100 for 15 min at room temperature. The cells were then incubated with blocking buffer (3% bovine serum albumin, 0.05% Triton X-100 in PBS) for 1 h and then reacted with primary antibodies diluted in blocking buffer for 1 h at room temperature. After reaction, excess primary antibodies were removed by immersing the coverslips in wash buffer (1.5% bovine serum albumin, 0.05% Triton X-100 in PBS) three times at 5 min intervals. Secondary antibodies conjugated with Alexa Fluor 568 or 488 (Thermo Fisher) were added to the cells, which were incubated in the dark for 1 h at room temperature. The cells were then soaked in the wash buffer three times at 5 min intervals and incubated with PBS containing Hoechst 33342 for 5 min at room temperature to stain the nuclei. Coverslips were mounted using Prolong Gold antifade reagent (Life Technologies). Fluorescence was detected using an ECLIPSE TE2000-U inverted microscope (Nikon, Tokyo, Japan).

## Results

### Nup88 interacts with vimentin intermediate filament protein

**Fig. 1:**
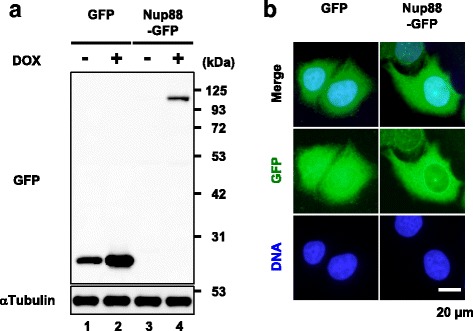
Overexpression of GFP-tagged Nup88 in HeLa cells. Stable transfectants harboring genes encoding *GFP* or *GFP-fused NUP88* under the control of a tetracycline-inducible system were incubated in medium without (−) or with (+) DOX for 2 days to stimulate protein overexpression. Overexpression and distribution pattern of the proteins in cells were assessed by immunoblotting (**a**) and by indirect immunofluorescence analysis (**b**), respectively

We attempted to establish stable cell lines expressing either GFP or GFP-tagged Nup88 (Nup88-GFP) as materials for screening Nup88 interacting proteins and for subsequent experiments. Because overexpression of Nup88 in HeLa cells is reported to induce multipolar spindles and the appearance of multinucleated cells [16], a tet-on expression system was applied in order to reduce both cell death and unexpected defects caused by Nup88 overexpression during the development of the stable cell lines. As shown in Fig. 1a, Nup88-GFP expression was undetectable in the absence of doxycycline (DOX). By contrast, GFP expression was strongly induced in the presence of DOX, indicating that the tet-on system worked well (Fig. 1a, lanes 3 and 4). When overexpressed, Nup88-GFP was localized to the nuclear rim and in the cytoplasm (Fig. 1b). A small portion of the protein was also found in the nucleus (Fig. 1b). Furthermore, overexpression of Nup88 induced multiple nuclei (Fig. 4b and c). These observations were consistent with previous reports [16, 19].

**Fig. 2:**
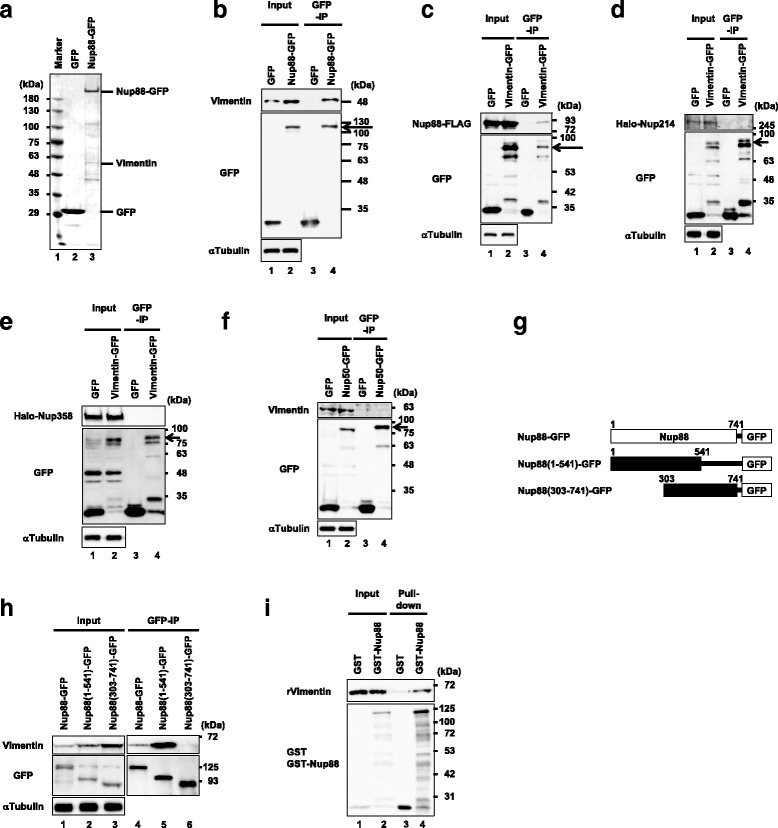
Vimentin is an interacting partner for Nup88. A GFP-coprecipitation assay was performed using cell lysates containing either GFP or GFP-tagged Nup88. Coprecipitates recovered were analyzed by SDS-PAGE and CBB protein staining (**a**). Specific interaction of vimentin with GFP-tagged Nup88 was confirmed by immunoblotting using an antibody against pan-vimentin. Inputs are 1.5% for vimentin and 1.0% for GFP constructs (**b**). GFP-coprecipitation assay for the vimentin interaction with Nup88 (**c**), Nup214 (**d**), Nup358 (**e**) and Nup50 (**f**). Inputs are 8.3% for both Nup88-FLAG and GFP constructs (**c**), 5.0% for Halo-tagged Nup214 (Halo-Nup214) and 3.3% for GFP constructs (**d**), 4.0% for Halo-tagged Nup358 (Halo-Nup358) and 3.0% for GFP constructs (**e**), and 3.7% for both GFP constructs and vimentin (**f**), respectively. Arrows indicate GFP-tagged vimentin (**c**–**e**) and GFP-tagged Nup50 (**f**), respectively. Schematic of GFP-tagged Nup88 constructs used in Fig. 2h. Amino acid residues of Nup88 are numbered (**g**). Determination of Nup88’s region responsible for vimentin binding. Cell lysates containing the GFP-tagged proteins described in Fig. 2g were analyzed. Inputs are 1.0% for GFP constructs and 1.3% for vimentin, respectively (**h**). GST-pulldown assay to examine the direct binding of recombinant GST-tagged Nup88 to recombinant vimentin. Inputs are 8.0% for both vimentin and GST constructs, respectively (**i**)

To screen Nup88 interacting proteins, we subjected the established cell lines to a GFP-coprecipitation assay. Cells expressing either GFP or Nup88-GFP upon DOX addition were extracted with NP-40 lysis buffer, and resultant lysates were incubated with anti-GFP antibody-conjugated agarose beads. Proteins recovered from the beads were separated by SDS-polyacrylamide gel electrophoresis and visualized with CBB (Fig. 2a). Proteins specifically recovered from Nup88-GFP bound beads were then subjected to mass spectrometry. As a result, vimentin, a type III IF protein, was obtained as a candidate Nup88 interacting protein (Fig. 2a lane 3). We then confirmed this interaction by an immunoblotting experiment using a vimentin specific antibody (Fig. 2b). In addition, the interaction was reproduced when lysates containing GFP-tagged vimentin (Vimentin-GFP) and FLAG-tagged Nup88 (Nup88-FLAG) were employed in the GFP-coprecipitation assay (Fig. 2c).

To address the specificity of vimentin binding to Nup88, we examined its binding to Nup214, Nup358, and Nup50. Nup214, which is localized on the cytoplasmic face of the NPC and forms a subcomplex with Nup88 [6], did not coprecipitate with Vimentin-GFP (Fig. 2d). Similarly, Nup358, which is also on the cytoplasmic face of NPCs and interacts with Nup88 [7], did not coprecipitate (Fig. 2e). Moreover, we found no evidence that Nup50, a component of the nuclear basket of NPC [38], interacts with Vimentin-GFP (Fig. 2f). Taken together, these experiments indicated vimentin-binding to Nups is specific for Nup88.

To determine the region of Nup88 responsible for vimentin binding, we generated GFP-tagged N- or C-terminal fragments of Nup88 for use in a GFP-coprecipitation assay (Fig. 2g). Coprecipitation of Nup88-GFP and vimentin was observed (Fig. 2h lane 4). Moreover, the N-terminal 541 aa residues of Nup88 (Nup88(1-541)-GFP) showed clear coprecipitation of vimentin, whereas the corresponding C-terminal 439 aa residues (Nup88(303-741)-GFP) did not (Fig. 2h lanes 4 and 5). These results suggested that the region of Nup88 spanning at least from 303 to 541 aa residues is involved in vimentin binding.

It has been reported that Nup88 does not bind to vimentin directly in an assay using purified recombinant proteins [39]. Given that we observed binding between Nup88 and vimentin using HeLa cell lysates, we reasoned the interaction might be indirect i.e., mediated by other proteins. To examine whether the interaction is indirect or not, we performed a GST-pull down assay using purified recombinant GST-tagged Nup88 (GST-Nup88) and recombinant human vimentin, both of which were prepared from *E. coli* (Fig. 2i). In this assay, recombinant vimentin was incubated with GST or GST-Nup88 in the presence of glutathione-conjugated Sepharose beads, and the proteins recovered from the beads were then analyzed by an immunoblotting assay. The results of the experiments showed GST coprecipitated vimentin at a much reduced level, while GST-Nup88 coprecipitated at a much higher level than GST (Fig. 2i lanes 3 and 4). These observations indicated that the interaction between Nup88 and vimentin is direct in vitro.

### Vimentin is functionally associated with the formation of multiple nuclei caused by Nup88 overexpression

**Fig. 3:**
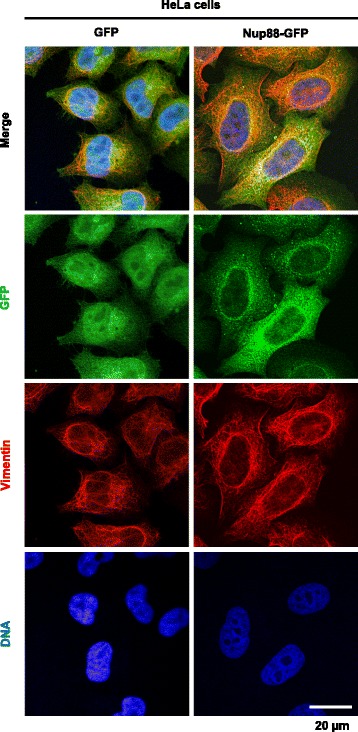
Analysis of colocalization of overexpressed Nup88 with vimentin. HeLa cell lines overexpressing GFP and GFP-tagged Nup88 were fixed with 4% paraformaldehyde and permeabilized with 0.5% Triton X-100. GFP constructs, vimentin, and nuclei were stained with rabbit anti-GFP antibody (green), mouse anti-vimentin antibody (red), and Hoechst 33,342 (blue), respectively

**Fig. 4:**
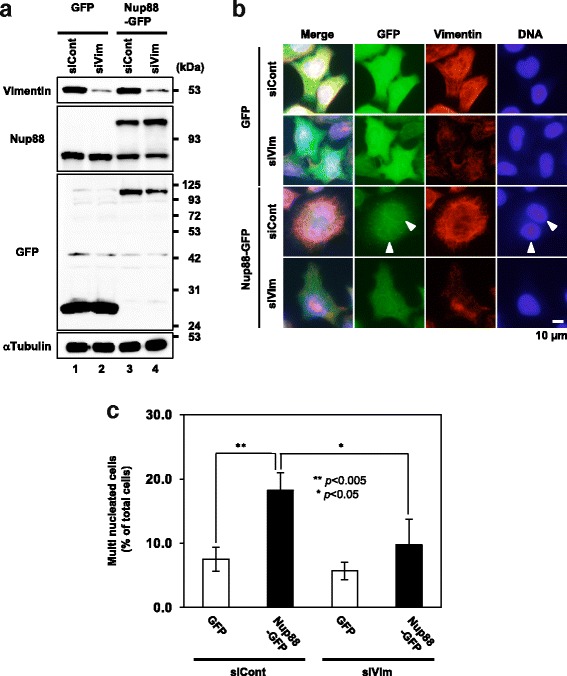
Depletion of vimentin by siRNA transfection suppresses the formation of multinuclei induced by Nup88 overexpression. HeLa cell lines treated with control siRNA (siCont) or vimentin-specific siRNA (siVim) were overexpressed with GFP and GFP-tagged Nup88 in the presence of DOX for 2 days. Depletion of vimentin was confirmed by immunoblotting (**a**) and immunofluorescence (**b**). Multinucleated cells were counted in images obtained from the indirect immunofluorescence assay. Data were collected from at least four independent experiments. Total cell number analyzed in the experiment for siCont-treated GFP expressing cells, siCont-treated Nup88-GFP expressing cells, siVim-treated GFP expressing cells, and siVim-treated Nup88-GFP expressing cells were 1296, 1156, 1377 and 1284 cells, respectively (**c**). White arrowheads indicate multinuclei (binuclei) (**b**). Error bars indicate ± SD (**c**)

In order to examine the in vivo association of Nup88 with vimentin, an indirect immunofluorescence assay was performed (Fig. 3). Overexpressed Nup88-GFP was clearly observed at the nuclear rim and in the cytoplasm and to a lesser extent in the nucleus. By contrast, vimentin was observed exclusively in the cytoplasm. Because overexpressed Nup88 in tumor tissues is observed in the cytoplasm [3], we anticipated that Nup88 would colocalize with vimentin in the cytoplasm. However, colocalization of Nup88-GFP and vimentin in the cytoplasm was unclear due to the widespread diffusion of Nup88-GFP, while evidence of colocalization was seen at or around the nuclear rim (Fig. 3). We therefore switched the experiment to analyze the functional characterization of the interaction on Nup88-dependent multinuclei formation by vimentin depletion (Fig. 4). An immunoblotting assay revealed that vimentin specific siRNA transfection resulted in an approximately 70% reduction of vimentin expression without affecting the endogenous expression of Nup88 in both GFP and Nup88-GFP expressing cells (Fig. 4a). An indirect immunofluorescence assay also confirmed the depletion of vimentin (Fig. 4b). We then examined the effect of vimentin depletion on multiple nuclei formation by counting cells containing multinuclei (Fig. 4b). Control transfectants expressing GFP or Nup88-GFP showed the proportion of multinucleated cells to be 7.5 and 18.2%, respectively, while vimentin siRNA transfectants of these cell lines showed the proportion of multinucleated cells to be 5.7 and 9.8%, respectively. These results suggested a functional association between vimentin and the formation of multinuclei.

### Nup88 protects phospho-vimentin from dephosphorylation through interacting with the N-terminal region of vimentin

**Fig. 5:**
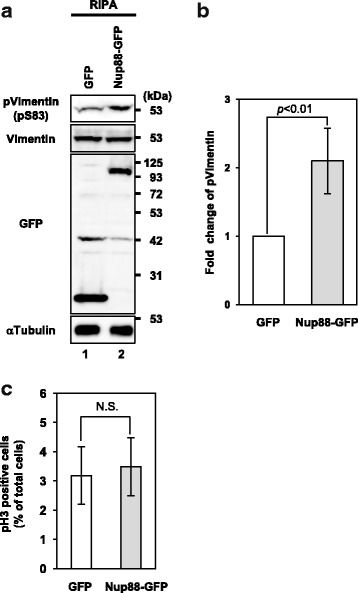
Increased expression of phospho-vimentin in Nup88 overexpressing cells. Phospho-vimentin expression was analyzed in HeLa cells overexpressing GFP and Nup88-GFP by immunoblotting using antibodies against pan-vimentin, phospho-vimentin (pS83), GFP and α-tubulin (**a**). Relative expression of phospho vimentin in cells expressing GFP and Nup88-GFP (**b**). Data were collected from three independent experiments including the result shown in Fig. 5a (**b**). Cells in mitosis were monitored by an immunofluorescence assay. HeLa cells overexpressing GFP or GFP-tagged Nup88 were fixed and stained with antibodies against phospho-histone H3 (pH 3) to distinguish mitotic cells from interphase cells. Data were collected from six independent experiments. Error bars indicate ± SD (**b** and **c**). N. S. means not significant (**c**)

The phosphorylation status of vimentin is critical for its organization and function [20]. We therefore examined the phosphorylation status of vimentin by monitoring phospho-Ser83 (pS83), which is phosphorylated by Plk1 during mitosis [30]. The phosphorylation of Ser83 in Nup88-GFP expressing cells was approximately 2-fold higher than that in GFP expressing cells, even though total vimentin expression was comparable between these cell lines (Fig. 5a and b). It is reported that vimentin IF is phosphorylated predominantly in mitosis [30–32] and that overexpression of Nup88 induces a mitotic defect in HeLa cells [16]. We therefore considered the possibility that the increased level of pSer83 is due to cell cycle delay or arrest in M phase. To estimate the ratio of cells in M phase, an indirect immunofluorescence assay using anti-phospho-Histone H3 was performed. As shown in Fig. 5c, the ratio was similar between GFP and Nup88-GFP expressing cells. Thus, these results indicated that the increase of phospho-vimentin in Nup88-GFP expressing cells was not due to arrest or delay of M phase.

**Fig. 6:**
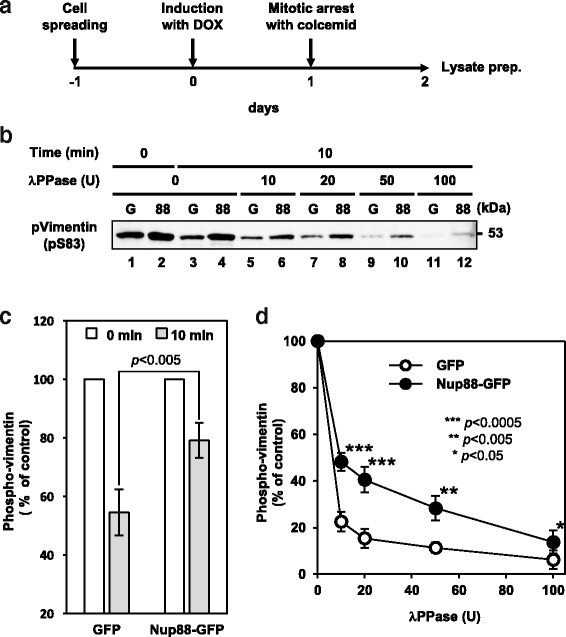
Overexpression of Nup88 inhibits vimentin dephosphorylation. Schematic of cell lysates preparation from cells arrested in mitosis (**a**). Mitotic cell lysates containing GFP or Nup88-GFP were incubated in the absence and presence of λPPase for 10 min, followed by detecting phospho-vimentin (pVimentin) by immunoblotting assay (**b**). Decrease of pVimentin in the absence of λPPase corresponding to Fig. 6b lanes 1-4 (**c**). A decrease of pVimentin in the presence of several units of λPPase corresponding to Fig. 6b lanes 3-12 (**d**). Data were collected from four independent experiments (**c** and **d**). Error bars indicate ± SD of four experiments (**c** and **d**)

The results shown in Fig. 5 raised the question of how Nup88 increases the level of phospho-vimentin. Because Nup88 was found to bind to vimentin directly (Fig. 2i), we considered two possibilities for the observed increase in phospho-vimentin as follows; either (i) Nup88 recruits kinases to vimentin or (ii) the interaction with Nup88 sterically prevents vimentin from being dephosphorylated by phosphatases. To examine the latter possibility, we first prepared cell lysates from GFP and Nup88-GFP expressing cells arrested in metaphase by colcemid treatment (Fig. 6a) before confirming the abundance of phospho-vimentin (Fig. 6b lanes 1 and 2). We subsequently analyzed the effect of Nup88 on endogenous phosphatases to monitor the decreased levels of phospho-vimentin. By comparison to the 0 min control (Fig. 6b lanes 1 and 2), phospho-vimentin levels were reduced by incubation for 10 min in either cell lysate (Fig. 6b lanes 3 and 4), suggesting that endogenous phosphatases were active under these experimental conditions. In addition, statistical analysis revealed that the decrease of phospho-vimentin in lysates containing Nup88-GFP was significantly lower than in lysates containing GFP (Fig. 6c). To further examine whether the inhibitory effect was specific to endogenous phosphatases or not, we assessed the phosphorylation status of vimentin in the presence of several units of bacteriophage lambda protein phosphatase (λPPase). When cell lysates were incubated with λPPase, phospho-vimentin levels decreased in a manner dependent on λPPase activity (Fig. 6b, lanes 5-12). Phospho-vimentin in lysates containing Nup88-GFP was significantly more resistant to λPPase than in lysates containing GFP (Fig. 6d). These findings suggest that the protection of phospho-vimentin by Nup88 is non-specific to phosphatases.

**Fig. 7:**
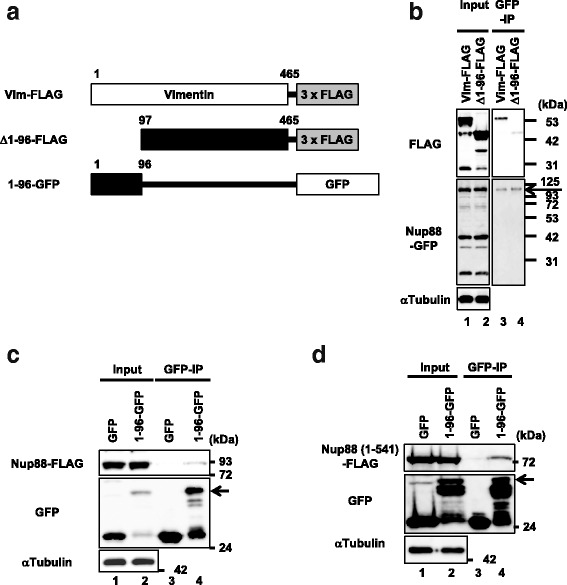
Overexpressed Nup88 interacts with the N-terminal region of vimentin. Schematic of vimentin constructs used in the GFP-coprecipitation assay (**a**). Nup88 overexpressing cells that transiently co-overexpress either full-length vimentin (Vim-FLAG) or an N-terminal truncated vimentin (Δ1-96-FLAG) were subjected to the GFP-coprecipitation assay (**b**). Cell lysates containing Nup88-FLAG with either control GFP or 1-96-GFP were subjected to the GFP-coprecipitation assay (**c**). Interaction between the Nup88 truncation and the vimentin N-terminal region (**d**). Inputs are 6.0% for FLAG constructs and 4.0% for Nup88-GFP (**b**), 0.7% for Nup88-FLAG and 1.3% for GFP constructs (**c**), and 2.0% for both Nup88(1-541)-FLAG and GFP constructs (D), respectively. Arrows indicate Nup88-GFP (**b**) and 1-96-GFP (**c** and **d**), respectively

There are a large number of phosphorylation sites located on the N-terminal 96 aa residues of vimentin [28, 29]. Because Nup88 protects vimentin from phosphatases (Fig. 6), we speculated that the interaction site for Nup88 is within the N-terminal region of vimentin. To examine this possibility, we performed a GFP-coprecipitation assay using lysates containing either full-length vimentin (Vim-FLAG) or a truncated version of the protein where the N-terminal 96 aa residues were missing (Δ1-96-FLAG) (Fig. 7a). Both constructs were expressed at similar levels in Nup88-GFP expressing cells (Fig. 7b lanes 1 and 2). Although Nup88-GFP was precipitated at similar levels in both cell lysates, coprecipitation of Vim-FLAG was 2.6-fold higher than that of Δ1-96-FLAG (Fig. 7b lanes 3 and 4). These findings suggested the N-terminal 96 aa residues of vimentin are required for its interaction with Nup88. To confirm its requirement more directly, either a GFP-tagged N-terminal vimentin fragment (1-96-GFP) or control GFP was co-overexpressed with Nup88-FLAG in HeLa cells, and then the cell lysates were employed in the GFP-coprecipitation assay. As a result, 1-96-GFP specifically coprecipitated with Nup88-FLAG (Fig. 7c). We further confirmed the binding of 1-96-GFP to FLAG-tagged Nup88(1-541), which contains the vimentin binding region (Figs. 2h and 7d). Taken together, these data indicated that the N-terminal 96 aa residues of vimentin are responsible for Nup88 binding.

## Discussion

Although recent studies have suggested that Nup88 overexpression can promote the early step of tumorigenesis by inducing formation of multinuclei, the molecular basis of how the multinucleated phenotype occurs is still poorly understood [16, 18]. To better understand the molecular mechanism, we screened Nup88-interacting proteins by a proteomic approach and identified vimentin as a novel Nup88 interacting protein (Fig. 1). Nup88 was found to interact with the N-terminal 96 aa residues of vimentin, which is a region of the protein predominantly phosphorylated in mitosis (Figs. 2 and 7). Moreover, this interaction with Nup88 protects phosphorylated vimentin from phosphatases (Fig. 4). Cells depleted in vimentin exhibited a decreased occurrence of multinuclei caused by Nup88 overexpression (Fig. 3).

It is reported that Nup88 binds to lamin A [39]. Interestingly, despite the fact that both lamin A and vimentin have a similar domain organization as IF proteins [23], lamin A is reported to bind to Nup88 through its C-terminal tail domain [39] whereas vimentin was found to interact with Nup88 through its N-terminal region (Fig. 7). Nonetheless, these two IF proteins bind to almost the same region of Nup88 (Fig. 2). Our findings imply that the mode of binding of Nup88 to vimentin is distinct from that of Nup88 to lamin A.

We found that cells overexpressing Nup88 exhibited increased levels of phospho-Ser83 within the N-terminal 96 aa of vimentin (Fig. 5) and that dephosphorylation at the site occurs more gradually in mitotic lysates containing an excess amount of Nup88 than in control lysates (Fig. 6). These results suggested that Nup88 inhibits phosphatases. Because the suppression was observed even in the presence of an unrelated phosphatase, it is unlikely that Nup88 actively and functionally inhibits such phosphatases (Fig. 6). Instead, rather than inhibiting the phosphatase directly, we propose a mechanism in which Nup88 masks the phosphorylated site and protects it from phosphatases. Moreover, our results show that Nup88 interacts with the N-terminal region of vimentin (Fig. 7).

The phosphorylation status of vimentin is critical for its organization and function [20]. We found that the depletion of vimentin suppressed Nup88-dependent multinuclei formation (Fig. 4). However, it is still unclear how vimentin participates in the phenotype (Fig. 3). It is reported that both Ser71 and Ser72 within the N-terminal region of vimentin are phosphorylated by Rho-associated kinases and Aurora B, respectively [33, 34]. Mutant vimentin, in which Ser71 and Ser72 cannot be phosphorylated by these kinases, is also reported to give rise to multinuclei and aneuploidy [34, 40, 41]. In the present study, we found that Nup88 could bind to the N-terminal region (Fig. 7) and protect phospho-Ser83 from phosphatases in HeLa cell lysates (Fig. 6). Moreover, a GST-pulldown assay using recombinant proteins purified from *E. coli* indicated that Nup88 could also bind to non-phosphorylated vimentin (Fig. 2i). It is therefore possible that Nup88 also masks the N-terminal region of vimentin against kinases as well as phosphatases. If this is the case, Nup88 may influence vimentin organization through protecting its N-terminal region from kinases, thereby promoting the multinucleated phenotype.

The specific regulation of vimentin organization during cancer development has remained unclear even though vimentin expression is correlated with malignant transformation of cancer [20]. Here, we found that Nup88 could affect the phosphorylation status of vimentin (Fig. 6). Our results also suggest that Nup88 affects vimentin organization by altering its phosphorylation status, which contributes to cancer malignancy. To pursue this line of research, it would be informative to examine the co-overexpression of Nup88 with vimentin in malignant tumor tissues.

## Conclusions

Nup88 can affect the phosphorylation status of vimentin, which may contribute to the Nup88-dependent multinucleated phenotype by changing the organization of vimentin.

## Abbreviations

CMV: Cytomegalovirus
DOX: Doxycycline
GFP: Green fluorescent protein
IF: Intermediate filament
NP-40: Nonidet-P40
NPC: Nuclear pore complex
Nups: Nucleoporins
RIPA: Radio immunoprecipitation assay
siRNA: Small interference RNA
